# Acute promyelocytic leukemia current treatment algorithms

**DOI:** 10.1038/s41408-021-00514-3

**Published:** 2021-06-30

**Authors:** Musa Yilmaz, Hagop Kantarjian, Farhad Ravandi

**Affiliations:** The Department of Leukemia, MD Anderson Cancer Center, Houston, Texas USA

**Keywords:** Chemotherapy, Acute myeloid leukaemia

## Abstract

In 1957, Hillestad et al. defined acute promyelocytic leukemia (APL) for the first time in the literature as a distinct type of acute myeloid leukemia (AML) with a “rapid downhill course” characterized with a severe bleeding tendency. APL, accounting for 10–15% of the newly diagnosed AML cases, results from a balanced translocation, *t*(15;17) (q22;q12-21), which leads to the fusion of the promyelocytic leukemia (*PML*) gene with the retinoic acid receptor alpha (*RARA*) gene. The PML–RARA fusion oncoprotein induces leukemia by blocking normal myeloid differentiation. Before using anthracyclines in APL therapy in 1973, no effective treatment was available. In the mid-1980s, all-trans retinoic acid (ATRA) monotherapy was used with high response rates, but response durations were short. Later, the development of ATRA, chemotherapy, and arsenic trioxide combinations turned APL into a highly curable malignancy. In this review, we summarize the evolution of APL therapy, focusing on key milestones that led to the standard-of-care APL therapy available today and discuss treatment algorithms and management tips to minimize induction mortality.

## Introduction

Acute promyelocytic leukemia (APL), a subtype of acute myeloid leukemia (AML), accounts for 10–15% of newly diagnosed AML cases. Approximately 800 patients are diagnosed with APL every year in the United States [1]. It often presents with abnormal white blood count (WBC) levels, low platelets, coagulopathy, and bleeding that require a prompt diagnosis and treatment.

APL results from a balanced translocation, commonly *t*(15;17) (q22;q12-21), which leads to the fusion of the promyelocytic leukemia (*PML*) gene with the retinoic acid receptor alpha (*RARA*) gene [2]. In about 10% of the cases, a successful cytogenetic analysis may lack classic t(15;17). In the majority of such cases, a molecular analysis nevertheless reveals an underlying *PML–RARA* fusion transcript formed as a result of cytogenetically cryptic or complex insertion events [2]. In other cases, less commonly, rearrangements of 17q21 lead to the fusion of *RARA* to alternative partner genes such as *NPM* (nucleophosmin), *PLZF* (promyelocytic leukemia zinc finger), and *NuMA* (nuclear mitotic apparatus) associated with *t*(5;17)(q35;q12-21), *t*(11;17)(q23;q21), and *t*(11;17)(q13;q21), respectively. The resulting *RARA* fusion product can form homodimers and disrupt normal *RARA* signaling [3]. It binds to retinoic acid response elements of target genes and recruits co-repressors such as DNA methyltransferases and histone deacetylases, and sequesters retinoic X receptor and the wild-type PML protein, which finally leads to suppression of genes necessary for granulocytic differentiation [4].

In 1957, Hillestad et al. defined acute promyelocytic leukemia (APL) for the first time in the literature as a distinct clinical entity with a “rapid downhill course” characterized with a severe bleeding tendency [5]. Anthracycline monotherapy was first successfully used in APL in 1973 [6]. Induction therapy with anthracyclines improved response rates (55–88%) and survival in APL; however, these improvements were suboptimal [6, 7]. Accumulation of undifferentiated promyelocytes in the bone marrow enabled the empirical use of differentiation therapy years before APL molecular targets were identified [8]. Sachs et al. discovered that leukemia cells could be prompted to differentiate [9]. In the early 1980s, all-trans-retinoic acid (ATRA) was shown to induce functional and morphological maturation in APL cells [10, 11]. ATRA causes a conformational change of the *PML–RARA* fusion transcripts, leading to the release of the co-repressors, recruitment of histone acetyltransferases, and relief of transcriptional repression, which causes the treated APL cells to undergo terminal myeloid differentiation and finally apoptosis [12].

**Fig. 1:**
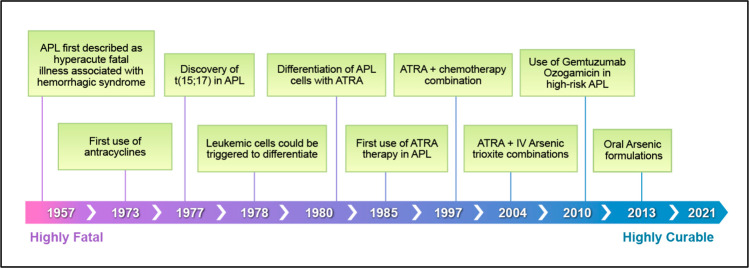
Evolution of therapy in APL. Acute promyelocytic leukemia milestones.

In the mid-1980s, ATRA was used in patients with APL, resulting in high response rates (>90%) [13, 14]. With ATRA monotherapy, the duration of response was usually short, 3–6 months. Later, the development of ATRA and chemotherapy combinations allowed APL to become a highly curable disease (Fig. 1).

## Approach to suspected APL

**Fig. 2:**
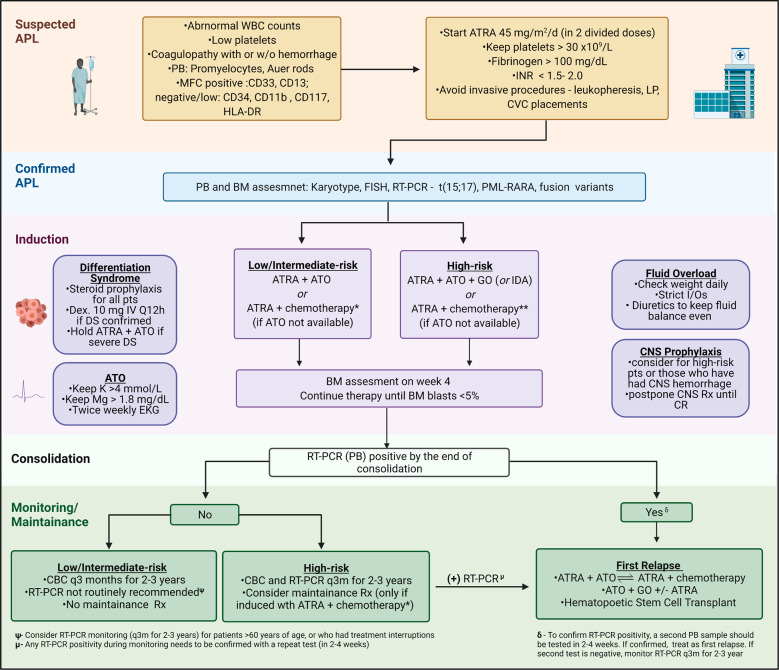
Approach to suspected APL and treatment algorithm. APL acute promyelocytic leukemia, WBC white blood cell count, PB peripheral blood, BM bone marrow, MFC multicolor flow cytometry, ATRA retinoic acid, LP lumbar puncture, CVC central venous catheter, DS differentiation syndrome, Dex dexamethasone, ATO arsenic trioxide, GO gemtuzumab ozogamicin, IDA idarubicin, I/Os intake and output, CNS central nervous system, RT-PCR reverse transcription polymerase chain reaction. *Lower risk APL: ATRA + IDA or ATRA + GO **Higher risk: ATRA + IDA or ATRA + DNR + ARA-C.

APL must be considered at the top of the differential diagnosis in patients with suspected leukemia and appropriate presentation, and every possible effort should be made to rule it out promptly (Fig. 2). APL usually presents at younger ages than non-APL AML; the median age at diagnosis is 40 vs. 70 years old [15]. Hispanic ethnicity and obesity have been reported as prevalent presentation features in APL [16, 17]. Considering these epidemiological data, an obese Hispanic patient (in her/his forties) may fit an APL stereotype. APL evaluation should start with a morphological examination of the peripheral blood. Nuclear morphology is characterized by eccentric, usually bilobed with a folded contour and with a prominent nucleolus. Microscopic identification of circulating promyelocytes with irregular azurophilic granules or Auer rods strongly supports APL diagnosis. A consumptive coagulopathy consisting of elevated partial thromboplastin time (PTT), prothrombin time (PT), and D-dimers, together with hypofibrinogenemia, and thrombocytopenia is another common feature present in three-quarters of the patients at diagnosis [15]. Clinical signs of coagulopathy vary from mild mucocutaneous bleeding to severe intracranial or pulmonary hemorrhage. On rare occasions, some patients may have concurrent deep-vein thrombosis or pulmonary emboli, or other thrombotic events [18].

In APL, most deaths occur within the first month of diagnosis [19–21]. According to the Swedish Acute Leukemia Registry data, approximately one-third of the patients die within 30 days of diagnosis, and 35% of the deaths occur before an ATRA dose [22]. Immediate administration of ATRA at the first suspicion of APL diagnosis is of extreme importance. Clinicians should establish a reliable system to provide fast ATRA delivery while the patient is still in the hospital emergency room. Similarly, molecular and cytogenetic testing should be obtained immediately upon presentation. If further testing rules out APL, ATRA can be discontinued with zero or minimal toxicity.

In addition to prompt ATRA administration, coagulopathy should be adequately corrected by keeping internationalized normalized ratio (INR) for PT at less than 1.5–2.0, fibrinogen greater than 100 mg/dL platelets greater than 30,000/µL through blood product transfusions. Invasive procedures, such as central line placements, lumbar punctures, and leukapheresis, should be avoided.

WBC count at presentation is a useful prognostic factor segregating patients into low-, intermediate-, and high-risk categories. Low- and intermediate-risk APL (differentiated by platelet counts above and below 40 × 10^9^/L) also referred to as standard-risk APL are defined by a WBC count of equal to or less than 10,000/µL. A presentation WBC count greater than 10,000/µL represents high-risk APL.

To minimize the early mortality in APL, Jillella et al. designed a prospective multicenter clinical trial facilitating APL comanagement strategy between academic institutions and community oncology practices [23]. This approach allowed use of remote consultancy between APL experts and community oncologists treating patients with APL. Developing a simplified algorithm that focused on prevention of early deaths, they were able to reduce early mortality to 8.5%. These promising results have led to a national intergroup clinical trial (NCT#03253848) “Simplified Patient Care Strategy in Decreasing Early Death in Patients With Acute Promyelocytic Leukemia” that is currently in progress.

## ATRA plus chemotherapy

After the role of ATRA monotherapy in APL was established, several phase III randomized clinical trials explored whether induction therapy with ATRA is superior to chemotherapy alone (Table 1). In the North American Intergroup Protocol study (I0129), 346 patients with newly diagnosed APL were randomized to receive ATRA monotherapy or daunorubicin (DNR) and cytarabine (ARA-C) as induction [24]. All patients who had a complete remission (CR) received two consolidation cycles consisting of DNR + ARA-C. Although patients in both arms achieved similar CR rates after induction (72% vs. 69%), the 3-year disease-free survival (DFS) and overall survival (OS) rates were significantly superior in ATRA arm than in the chemotherapy-alone arm, 67 vs. 32%, and 67 vs. 50%, respectively (Table 1). After consolidation therapy, patients in CR were randomly assigned to maintenance treatment with ATRA or observation. The authors demonstrated that maintenance therapy with ATRA significantly contributed to superior DFS and OS.

**Table 1:** Summary of select randomized clinical trials for patients with newly diagnosed acute promyelocytic leukemia.

Main objective	Induction/consolidation	Maintenance Rx	No. of Pts		CR rate	Induction death	DFS/EFS*	OS	Clinical trial
ATRA → CT vs. CT alone	ATRA → DNR + ARA-C	None		54	91%	9%	*79% at 1 y	91% at 1 y	European APL group (1993)
	DNR + ARA-C			47	81%	8%	*50% at 1 y	80% at 1 y	
ATRA → CT vs. CT alone	ATRA → DNR + ARA-C	ATRA vs. Observation		172	72%	11%	67% at 3 y	67% at 3 y	North American Intergroup (1997)
	DNR + ARA-C			174	69%	14%	32% at 3 y	50% at 3 y	
Sequential vs. concurrent ATRA with CT	ATRA → DNR + ARA-C	ATRA vs. 6-MP/MTX		109	95%	7%	77% at 2 y	81% at 2 y	European APL group (1999)
	ATRA + DNR + ARA-C			99	94%	6%	84% at 2 y	84% at 2 y	
ATRA + CT with ARA-C vs. without ARA-C	ATRA + DNR + ARA-C	ATRA + 6MP/MTX		95	99%	1%	*93% at 2 y	98% at 2 y	European APL group (2006)
	ATRA + DNR			101	94%	4%	*77% at 2 y	90% at 2 y	
ATRA + ATO vs. ATRA + CT	ATRA + ATO	None		127	100%	0%	*97% at 50 m	99% at 50 m	Italian–German APL0406 (2016)
	ATRA + IDA	ATRA + 6MP/MTX		136	97%	3%	*80% at 50 m	93% at 50 m	
ATRA + ATO vs. ATRA + CT	ATRA + ATO + GO^a^	None		116	94%	4%	97% at 4 y	93% at 4 y	UK AML working group (2015)
	ATRA + IDA	none		119	89%	6%	78% at 4 y	89% at 4 y	

*DNR* daunorubicin, *ARA-*C cytarabine, *IDA* idarubicin, *CT* chemotherapy, *ATO* arsenic trioxide (IV), *MTX* methotrexate, *6-MP* mercaptopurine, *DFS* disease free survival, *EFS* event free survival, *OS* overall survival, *y* year, *m* month.

*EFS, → sequential, **+** concurrent.

^a^GO (gemtuzumab ozogamicin) only for high risk patients.

Although sequencing ATRA with chemotherapy improved outcomes, approximately one-third of the patients still relapsed. To explore the efficacy of concurrent versus (vs) sequential use of ATRA and chemotherapy, the Europen APL group randomized patients with newly diagnosed APL (age <65 years old, WBC < 5000) to ATRA followed by DNR + ARA-C (ATRA → CT) vs. ATRA plus DNR + ARA-C (ATRA + CT) [25]. The CR rates, 2-year EFS, and OS rates were comparable, 95 vs. 94%, 77 vs. 84%, and 81 vs. 84% in ATRA → CT and ATRA + CT arms, respectively. However, 2-year relapse rates were more favorable for concurrent ATRA + CT (6%) than for sequential ATRA → CT (16%), *p* = 0.04. Maintenance therapy with ATRA or 6-MP/methotrexate (MTX) significantly decreased relapse rates and improved survival.

The combination of ATRA plus antracycline appears to be equally effective in curing APL as when antracylines are combined with ARA-C and ATRA. Several study groups, particularly the Spanish PETHEMA group (in LPA 96 and 99 clinical trials [26, 27]), reported high CR rates and low relapse rates in patients with newly diagnosed APL treated with ATRA and IDA combination without ARA-C. However, the European APL group designed a study randomizing patients with newly diagnosed APL (age <60 years old, WBC < 10,000) to receive ATRA plus DNR with (*N* = 95) or without ARA-C (*N* = 101) [28]. In the ARA-C and the no ARA-C groups, the CR, the 2-year cumulative incidence of relapse, EFS, and OS rates were 99 vs. 94% (*p* = 0.12), 5 vs. 16% (*p* = 0.01), 93 vs. 77% (*p* = 0.002), and 98 vs. 90% (*p* = 0.006), respectively. However, it is difficult to compare these data to the data published by the PETHEMA group due to differences in anthracyclines used and their total cumulative dose. Furthermore, patients in the PETHAMA trials received ATRA during consolidation. A study by Burnett et al. (conducted by the MRC in the United Kingdom) randomized newly diagnosed patients with APL (age <60 years old) to ATRA plus DNR/ARA-C/etoposide vs. ATRA plus IDA (the PETHEMA regimen without ARA-C) [29]. No difference in CR rates (91 vs. 93%) and 4-year OS rates (81 vs. 84%) was reported, and less myelosuppression was seen in patients who received the regimen omitting ARA-C. This study suggests that most APL patients can be cured without ARA-C, and perhaps with less toxicity.

## ATRA plus arsenic trioxide

Although its mechanism of action is not fully understood, arsenic trioxide (ATO, As_2_O_3_) has dose-dependent dual effects on APL cells by preferentially inducing apoptosis (at high concentrations) and differentiation (at low concentrations). ATO monotherapy was investigated initially in patients with relapsed or refractory APL and found to induce CR in more than 80% [30, 31]. Several investigators (outside of the United States) studied the role of ATO monotherapy in patients with newly diagnosed APL. The CR, EFS, and OS (two or more years) rates were between 86 and 91%, 64 and 82%, and 88 and 91%, respectively [32–34], suggesting that 20–30% of the patients still experience disease relapse. In general, it has been shown that fewer cycles of ATO administered are associated with higher likelihood of relapse [35].

Preclinical studies demonstrated significant synergism between ATRA and ATO via induction of cell differentiation and apoptosis in promyelocytic leukemia [36, 37]. Furthermore, several early-phase clinical trials reported favorable results using ATRA plus ATO combination in patients with APL [38, 39]. In a pilot study by Estey et al. [39] at MD Anderson Cancer Center (MDACC), 44 patients with newly diagnosed APL were treated with ATRA plus ATO (gemtuzumab ozogamicin added in high-risk cases), resulting in an overall CR rate of 88% with no relapse in low-risk patients. In a later update of this clinical trial with an enrollment of a total of 82 patients (all risk categories), the CR and 3-year OS rates were 92% and 85%, respectively [40]. Favorable outcomes shown with this chemotherapy-free regimen paved the way for randomized phase III clinical trials.

In a multicenter and prospective noninferiority clinical trial (APL0406), 263 patients with newly diagnosed APL (low/intermediate-risk) were randomized to ATRA + ATO or ATRA + idarubicin (IDA) [41]. The CR and induction death rates were comparable in ATRA + ATO and ATRA + IDA arms, 100 vs. 97%, and 0 vs. 3%, respectively (Table 1). In a later update, the 50-month EFS and OS rates were 97 vs. 80%, 99 vs. 93%, respectively (*p* < 0.001 and *p* = 0.007) [42]. Patients in ATRA + ATO arms received no maintenance therapy, and patients in ATRA + IDA arm received ATRA + 6-MP + MTX maintenance (up to two years). Despite lack of maintenance therapy, the 50-month cumulative incidence of relapse rate was remarkably lower in ATRA + ATO group than in ATRA + IDA group, 2 vs. 14% (*p* = 0.001), respectively. In the most recent update, with a follow-up of 72 months, Cicconi et al. reported increased advantage of ATRA + ATO over time compared with ATRA + IDA [43]. A similarly designed phase III randomized clinical trial (AML17) demonstrated comparable outcomes for newly diagnosed patients with APL who received ATRA + ATO, showing lower relapse rates and better survival than ATRA + IDA [44] (Table 1). However, it is important to note that ATO dose schedules used in these phase III clinical trials were different: APL0406 used a ATO + ATRA schedule based on MDACC regimen [39] (ATO IV 0.15 mg/kg/day daily until CR, and 0.15 mg/kg/day five days/week for four weeks of consolidation cycles 1–4), AML17 (ATO IV 0.3 mg/kg on days 1–5 of each cycle, and at 0.25 mg/kg twice weekly in weeks 2–8 of cycle one and weeks 2–4 of cycles 2–5). ATO dose schedule used in AML17 trial may be considered more “user friendly” as it is given only twice weekly (compared with five days per week) during consolidation cycles. However, irrespective of how ATO was administered, both APL0406 and AML17 trials have shown similar results. Hence, ATRA plus ATO, a chemotherapy-free regimen, has become the standard treatment of choice for non-high-risk patients with APL (Fig. 2).

The optimal regimen for patients with high-risk APL remains a debated issue. These patients have a higher possibility of induction mortality due to increased risk of fluid overload, differentiation syndrome, respiratory failure, disseminated intravascular coagulation (DIC), and severe bleeding. Hence, controlling leukocytosis and treating DIC early on is critical. IDA and gemtuzumab ozogamicin (GO) are the most commonly used antineoplastic agents to control leukocytosis in high-risk APL patients who are induced with a chemotherapy-free regimen such as ATRA + ATO. In a study by Australasian Leukaemia and Lymphoma Group (APML4), IDA was added to ATRA + ATO regimen to treat patients with high-risk APL [45]. IDA was administered on days two, four, six, and eight during induction. The CR, early mortality, 5-year DFS, and OS rates were 91, 9, 95, and 87%, respectively.

GO, an anti-CD33 monoclonal antibody conjugated to calicheamicin, is another drug that has been studied in high-risk APL as adjunctive therapy to ATRA + ATO regimen **(**Fig. 2**)**. In prospective ATRA + ATO clinical trials performed by MD Anderson and UK AML working group, GO was administered during induction for patients with WBC greater than 10,000 at diagnosis [40, 44, 46]. In both clinical trials, patients continued ATRA + ATO during consolidation without GO, and none of the patients received maintenance therapy. In the MD Anderson study, which included 54 patients with high-risk APL, the early mortality rate was 4%, and the 5-year DFS and OS rates were 89% and 86%, respectively [46]. In the UK study, the 4-year OS rate was 89% in patients with the high-risk disease [44]. The Eastern Cooperative Oncology group (ECOG) and the Southwest Oncology group (SWOG) investigated a different treatment program for patients with newly diagnosed high-risk APL. Although induction therapy was similar, using GO plus ATRA + ATO, consolidation cycles consisted of ATO monotherapy (two cycles), followed by ATRA + DNR (two cycles), followed by GO monotherapy (two cycles) and included an ATRA + 6-MP + MTX maintenance (up to a year) [47]. The CR, 3-year DFS, and OS rates were 85%, 93%, and 88%, respectively.

In summary, added to ATRA plus ATO-based induction regimens, GO allows long-term OS rates close to 90% in patients with high-risk APL (Fig. 2). In the absence of GO, IDA can be used as an alternative for patients with normal left ventricular ejection [46].

## Maintenance therapy in APL

In the era of ATO, maintenance therapy is no longer needed in APL. In the largest randomized clinical trials, such as AML17 by the UK AML working group and APL0406 by the Italian–German study group, patients treated with ATRA plus ATO received no maintenance therapy and relapse after consolidation was exceedingly rare (Table 1). In SWOG/ECOG/Cancer and Leukemia Group B (CALBG) S0521 trial, 105 patients with APL who achieved molecular CR (ATRA + DNR + ARA-C induction, followed by two cycles of ATO monotherapy, followed by two cycles of DNR + ARA-C) were randomized to maintenance (ATRA + 6-MP + MTX) or no maintenance (observation) therapy, and no relapses occurred in either arm [48]. In AIDA 0493 protocol, patients who achieved molecular CR following induction (ATRA + IDA) and consolidation (CT without ATRA or ATO) were randomized into four arms: (1) 6-MP + methotrexate, (2) ATRA alone, (3) ATRA alternating with 6-MP and methotrexate, and (4) no therapy [49]. No DFS difference was observed at 12-years among maintenance-therapy recipients: 70, 69, 68, and 69%, respectively. Maintenance therapy is not part of our standard practice for patients who achieve molecular CR at the end of consolidation with ATRA plus ATO based regimens. However, maintenance therapy should be considered in high-risk patients who were treated with ATRA plus chemotherapy (without ATO).

## Central nervous system prophylaxis

Rarely seen in lower-risk APL, central nervous system (CNS) involvement occurs mostly in patients with high WBC count at presentation and those with CNS bleeding. Intrathecal (IT) chemotherapy has not been employed in major clinical trials investigating ATRA + ATO combination therapy. In addition, ATO is known to cross the blood–brain barrier and achieve therapeutic levels in cerebrospinal fluid [50, 51]. In a recent report, of the 187 patients with newly diagnosed APL who received ATRA + ATO induction (without IT prophylaxis), five patients with high-risk APL relapsed, among them three had CNS involvement [46]. Given its rare incidence and absence of significant data to support the use of IT therapy in ATRA + ATO era, universal CNS prophylaxis is not recommended. If CNS prophylaxis is to be employed, it should be limited to the high-risk patients or those with a CNS, retinal, or paraspinal hemorrhage, and should only be performed after achievement of CR and resolution of coagulopathy [52, 53].

## Common complications in APL

### Differentiation syndrome

Formerly known as ATRA syndrome, differentiation syndrome (DS) is a potentially life-threatening complication that usually emerges during the first days or weeks of APL therapy. Dyspnea, pulmonary infiltrates, pleural effusion, fever, weight gain, peripheral edema, hypotension, and acute renal failure are the hallmarks of this syndrome. A high-confidence diagnosis of DS is usually not possible due to the frequent incidence of other mimicker clinical conditions such as bleeding, infection, sepsis, or fluid overload. Hence prophylactic corticosteroids have been used in prospective clinical trials [40, 41, 45] and recommended to prevent DS for all patients with newly diagnosed APL, particularly in patients with high-risk disease [54]. Various doses, schedules, and formulations were used, such as methylprednisone 50 mg/day between days one and five, followed by tapering on day six or prednisone 0.5–1 mg/kg/day from day1 until CR [40, 41, 45]. Dexamethasone 10 mg (every 12 hours) should be administered for the treatment of suspected or overt DS, until resolution of symptoms and signs or for a minimum of three days [53]. ATRA plus ATO therapy should be held in patients with severe DS, characterized by detecting three or more clinical signs or symptoms [55].

### Unrecognized fluid overload

Treating APL patients with ATRA plus ATO for approximately two decades, we have noticed an important yet unrecognized clinical entity: fluid overload [52]. Commonly confused with DS, fluid overload can occur in the absence of DS. The etiology of fluid overload in APL can be explained in part by the capillary leakage caused by endothelial injury and, in part, by the large quantities of blood product infusions required to treat coagulopathy. In a recent study, 26 of 187 (14%) patients with newly diagnosed APL were reported to develop clinically significant fluid overload (10% or more weight gain) during ATRA + ATO induction [56]. A median of 4.5 liters of blood products was infused per patient during the induction course. In the multivariate analysis, a weight increase of 10% or more during induction was significantly associated with intensive care unit (ICU) admissions (due to hypoxia) and endotracheal intubations. Physical exam findings are usually subtle. However, if monitored carefully, gradual weight gain with positive fluid balance (daily intake/output) can easily be recognized. If patients are permitted to have a daily positive fluid balance, this can lead to hypoxia, eventual ICU transfer, and endotracheal intubation. Although the general notion is to use diuretics in patients with symptoms (hypoxia, peripheral edema), throughout APL induction, we advocate preemptive use of diuretics intending to keep the fluid balance even.

### Coagulopathy

Complex coagulopathy in APL consists of consumptive coagulation and fibrinolysis, resulting in major bleeding complications. Platelet counts and coagulation parameters, including PTT, PT, as well as fibrinogen levels, should be monitored daily to keep these parameters within range. However, it is essential to maintain an even fluid balance and avoid transfusion-associated fluid overload while correcting coagulopathy.

### Other treatment-related toxicities

Compared with chemotherapy, an ATO-based regimen is less likely to cause grade 3/4 neutropenia and thrombocytopenia. Particularly during consolidation cycles, less than 5% of the ATRA + ATO-treated patients experience grade 3/4 cytopenia compared with up to 50% of the ATRA + chemotherapy recipients [41]. ATO associated neutropenia may occur as a result of delayed bone marrow evaluation and unnecessarily prolonged ATO therapy. On the other hand, prolonged QTc and grade 3/4 hepatotoxicity is more common in ATRA + ATO than ATRA + chemotherapy, 13% vs. 0% (*p* < 0.01), and 57% vs. 5% (*p* < 0.01), respectively [41]. Both are managed with temporary discontinuation and dose modification of ATO. To lessen the risk of QTc prolongation, electrolytes (potassium, magnesium, and calcium) need to be monitored closely and corrected. Leukocytosis (>10,000) is observed in approximately half of the cases during ATRA + ATO induction therapy. It can effectively be controlled by hydroxyurea, but preferably by GO or a dose of an anthracycline [54]. Leukopheresis does not improve the outcome of patients presenting with hyperleukocytosis and should be avoided [54, 57].

## Relapsed APL

Overall, 5–10% of the patients with APL develop relapsed and/or refractory (R/R) disease. Most of the relapses occur within the first three years, and late relapses beyond three to four years are very rare [58]. Several regimens, such as ATRA, anthracyclines, ATO, high-dose ARA-C, and GO, have been used to treat R/R APL. The choice of chemotherapy depends on the regimen used for induction and whether the relapse occurred during therapy. Several studies have demonstrated high CR rates following ATO therapy in patients with R/R APL [59, 60]. In a phase II study, 35 patients with R/R APL were treated with ATO, followed by autologous stem cell transplantation. Following induction, 21 achieved CR (60%), and eventually 23 were able to undergo autologous stem cell transplant. The 5-year EFS and OS rates were 65% and 77%, respectively. Studies investigating the impact of combining ATO therapy with other agents have reported equivocal results. In a small study, 20 patients with R/R APL were randomized to receive ATO monotherapy or ATRA + ATO [61]. After one cycle of ATO with or without ATRA, the CR rates were the same, 80%, for both groups, suggesting no benefit of adding ATRA. Another small pilot study demonstrated the benefit of adding GO as consolidation after ATO induction [62]. Where available, GO may effectively control disease in APL patients who have had a molecular relapse.

Tamibarotene, a synthetic retinoid with superior differentiating activity compared with ATRA, is another viable option for multiple R/R APL. In a phase II study, single agent tamibarotene was shown to generate 64% overall response rate in R/R APL patients with at least two lines of prior therapies (including ATRA + ATO) [63]. However, event-free survival was still short.

## Secondary APL

Secondary APL (sAPL) is defined as APL emerging after chemotherapy and/or radiotherapy used for malignant or nonmalignant conditions and represents about 10% of the newly diagnosed APL cases [64]. Several retrospective studies demonstrated that the clinical characteristics and survival of patients with de novo APL and sAPL are comparable [65, 66]. In a prospective study by the French–Belgian–Swiss APL group, Braun et al., confirmed that patients with sAPL have clinical features and outcomes (CR rate, cumulative incidence of relapse, and OS rate) similar to those of patients with de novo APL [67].

## Oral arsenic formulations

**Fig. 3:**
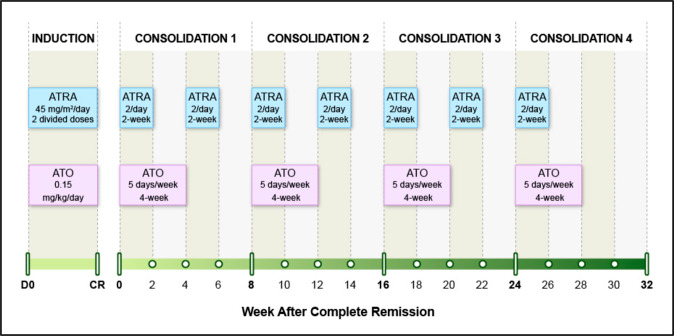
ATRA plus ATO treatment schedule. ATRA retinoic acid, ATO arsenic trioxide, mg milligram, 2/day two divided doses.

ATO, the backbone of APL therapy, is administered as an intravenous (IV) infusion, which necessitates daily visits to the chemotherapy infusion center during consolidation (five days per week for a total of 16 weeks over a period of eight months) (Fig. 3). To alleviate the cost and inconvenience associated with IV therapy, several groups developed oral arsenic formulations.

A liquid oral ATO formulation was found to be sufficiently bioavailable in patients with hematologic malignancies [68]. In relapsed APL, the regimen was highly effective at 10 mg/day dose, demonstrating an efficacy comparable to IV arsenic [69]. In a pilot study from Hong Kong, 62 patients with newly diagnosed APL received ATRA + oral ATO (10 mg/day) induction (patients aged <70 received DNR as well), and all achieved CR (Table 2). Consolidation cycles consisted of either DNR + ARA-C (age <70 years old) or ATRA monotherapy (age ≥70 years old), with all receiving ATRA maintenance therapy [70]. With a median follow-up of 37 months, the 5-year leukemia-free and OS rates were both 94%. While the severity and incidence of hepatotoxicity, leukocytosis and rash were comparable to that of IV ATO, no grade 3/4 QTc prolonging was observed with this liquid oral formulation [68–70]. Oral ATO formulations (ORH-2014 and encapsulated oral ATO) were recently studied in an early-phase study and were found to be safe, highly bioavailable with an arsenic exposure comparable to IV ATO [71, 72]. Future studies comparing oral and IV ATO formulations are expected.

**Table 2:** Summary of clinical trials explored oral arsenic formulations for patients with newly diagnosed APL.

Clinical trial	Induction Rx	Maintainance Rx	No. of Pts	CR rate	Induction death	DFS/EFS*	OS	Reference (year)
Phase II, two arm, nonrandomized	ATRA + oral ATO	ATRA	62	100%	0%	94% at 5 y	94% at 5 y	Gill et al. (2019)
	ATRA + DNR	ATRA	37	100%	0%	87% at 5 y	97% at 5 y	
Phase III, two arm, randomized	ATRA + RIF	ATRA - > RIF	124	99%	1%	98% at 2 y	99% at 3 y	Zhu et al. (2013)
	ATRA + IV ATO	ATRA - > IV ATO	127	97%	3%	96% at 2 y	97% at 3 y	
Phase II, single arm	ATRA + RIF	none	20	100%	0%	100% at 4 y	100% at 4 y	Zhu et al. (2014)
Phase II, single arm	ATRA + RIF	none	20	100%	0%	89% at 3 y*	100% at 3 y	Zhu et al. (2018)
Phase III, two arm, randomized	ATRA + RIF	none	69	100%	0%	97% at 2 y*	100% at 2 y	Zhu et al. (2018)
	ATRA + IV ATO	none	36	94%	6%	94% at 2 y*	94% at 2 y	
Phase III, two arm, randomized	ATRA + RIF + MT	ATRA + 6MP + MTX	40	100%	0%	100% at 5 y*	100% at 5 y	Yang et al. (2018)
	ATRA + IV ATO + MT	ATRA + 6MP + MTX	42	100%	0%	100% at 5 y*	100% at 5 y	

RIF A tetra-arsenic tetra-sulfide (As4S4)-containing a compound named realgar-Indigo naturalis formula, ATO arsenic trioxide, IV intravenous, MT mitoxantrone, 6-MP mercaptopurine, MTX methotrexate.

* denotes “EFS”.

A tetra-arsenic tetra-sulfide (As4S4), containing a compound named realgar-Indigo naturalis formula (RIF), is another oral arsenic formulation that has been explored by the Chinese investigators in patients with APL (Table 2). In a phase III study, 242 patients were randomized to ATRA plus RIF (60 mg/kg/day) or IV ATO induction [73]. All patients received three cycles of consolidation chemotherapy followed by maintenance therapy that consisted of sequential use of ATRA with RIF or IV ATO for two years. The CR, 2-year DFS, and OS rates were comparable between treatment arms, 99 vs. 97%, 98 vs. 96%, and 99 vs. 97%. Patients in RIF and IV ATO arms experienced a similar incidence of grade 3/4 hepatotoxicity (10 vs. 12%) and DS (19 vs. 25%) during induction, respectively. Although the results with RIF + ATRA induction were positive, this study used chemotherapy during consolidation cycles.

Later, two pilot studies [74, 75] (single arm) examined ATRA plus RIF without chemotherapy (in newly diagnosed APL), in a schedule similar to chemotherapy-free ATRA + IV ATO (Fig. 3). Patients received ATRA plus RIF until CR followed by ATRA (2 weeks on, 2 weeks off) and RIF (4 weeks on, 4 weeks off) consolidation for 7 months (without maintenance). Both studies had a small patient size with short follow-ups. However, the reported outcomes were remarkable: 100% CR rate, no induction death, and 100% OS at three- and four years (Table 2).

Given these favorable data, chemotherapy-free ATRA plus RIF regimen was investigated in a multicenter, noninferiority clinical trial [76]. Zhu et al. randomized 109 patients with newly diagnosed APL (lower risk) to receive ATRA plus RIF or IV ATO until CR followed by ATRA plus RIF or IV ATO consolidation for seven months without any maintenance therapy (Table 2). The CR, 2-year EFS, and OS rates were 100 vs. 94%, 97 vs. 94%, and 100 vs. 94%, respectively, which suggests that the oral chemotherapy-free regimen might be an alternative to standard IV ATO-based therapy. In another randomized study, similar favorable outcomes with ATRA plus RIF regimen were reproduced in a pediatric population [77]. Overall, these data suggest that ATRA plus RIF is not inferior to ATRA plus IV ATO in patients with newly diagnosed APL. RIF has been approved and is being used for APL therapy in China.

## Conclusions

The introduction of ATO into APL therapy has changed the treatment landscape and allowed the development of a chemotherapy-free regimen with high success rates. GO, or IDA, should be added early during induction therapy in patients with high-risk disease. In the absence or unavailability of ATO, ATRA plus chemotherapy combinations represent a reasonable alternative. Despite this progress, induction mortality remains one of the main obstacles in APL therapy. Clinicians must initiate ATRA treatment (on the earliest suspicion), diagnose promptly, and monitor vigorously during therapy to minimize expected complications of APL. Several oral arsenic formulations are highly bioavailable and effective in patients with APL. Already approved in China, oral arsenic therapy needs to be further explored in the United States and Europe.

## References

[1] Yamamoto JF, Goodman MT (2008). Patterns of leukemia incidence in the United States by subtype and demographic characteristics, 1997–2002. Cancer Causes Control.

[2] Grimwade D, Lo Coco F (2002). Acute promyelocytic leukemia: a model for the role of molecular diagnosis and residual disease monitoring in directing treatment approach in acute myeloid leukemia. Leukemia..

[3] Nowak D, Stewart D, Koeffler HP (2009). Differentiation therapy of leukemia: 3 decades of development. Blood.

[4] Sirulnik A, Melnick A, Zelent A, Licht JD (2003). Molecular pathogenesis of acute promyelocytic leukaemia and APL variants. Best Pract Res Clin Haematol.

[5] Hillestad LK (1957). Acute promyelocytic leukemia. Acta Med Scand.

[6] Bernard J, Weil M, Boiron M, Jacquillat C, Flandrin G, Gemon MF (1973). Acute promyelocytic leukemia: results of treatment by daunorubicin. Blood.

[7] Fenaux P, Pollet JP, Vandenbossche-Simon L, Morel P, Zandecki M, Jouet JP (1991). Treatment of acute promyelocytic leukemia: a report of 70 cases. Leuk Lymphoma.

[8] de The H, Chen Z (2010). Acute promyelocytic leukaemia: novel insights into the mechanisms of cure. Nat Rev Cancer..

[9] Sachs L (1978). Control of normal cell differentiation and the phenotypic reversion of malignancy in myeloid leukaemia. Nature..

[10] Breitman TR, Selonick SE, Collins SJ (1980). Induction of differentiation of the human promyelocytic leukemia cell line (HL-60) by retinoic acid. Proc Natl Acad Sci USA.

[11] Breitman TR, Collins SJ, Keene BR (1981). Terminal differentiation of human promyelocytic leukemic cells in primary culture in response to retinoic acid. Blood.

[12] Tomita A, Kiyoi H, Naoe T (2013). Mechanisms of action and resistance to all-trans retinoic acid (ATRA) and arsenic trioxide (As2O 3) in acute promyelocytic leukemia. Int J Hematol.

[13] Huang ME, Ye YC, Chen SR, Chai JR, Lu JX, Zhoa L (1988). Use of all-trans retinoic acid in the treatment of acute promyelocytic leukemia. Blood.

[14] Daenen S, Vellenga E, van Dobbenburgh OA, Halie MR (1986). Retinoic acid as antileukemic therapy in a patient with acute promyelocytic leukemia and Aspergillus pneumonia. Blood..

[15] Cicconi L, Lo-Coco F (2016). Current management of newly diagnosed acute promyelocytic leukemia. Ann Oncol.

[16] Douer D (2003). The epidemiology of acute promyelocytic leukaemia. Best Pract Res Clin Haematol..

[17] Tedesco J, Qualtieri J, Head D, Savani BN, Reddy N (2011). High prevalence of obesity in acute promyelocytic leukemia (APL): implications for differentiating agents in APL and metabolic syndrome. Ther Adv Hematol..

[18] Breen KA, Grimwade D, Hunt BJ (2012). The pathogenesis and management of the coagulopathy of acute promyelocytic leukaemia. Br J Haematol.

[19] Jácomo RH, Melo RA, Souto FR, de Mattos ER, de Oliveira CT, Fagundes EM (2007). Clinical features and outcomes of 134 Brazilians with acute promyelocytic leukemia who received ATRA and anthracyclines. Haematologica.

[20] Park JH, Qiao B, Panageas KS, Schymura MJ, Jurcic JG, Rosenblat TL (2011). Early death rate in acute promyelocytic leukemia remains high despite all-trans retinoic acid. Blood.

[21] Chen Y, Kantarjian H, Wang H, Cortes J, Ravandi F (2012). Acute promyelocytic leukemia: a population-based study on incidence and survival in the United States, 1975–2008. Cancer.

[22] Lehmann S, Ravn A, Carlsson L, Antunovic P, Deneberg S, Möllgård L (2011). Continuing high early death rate in acute promyelocytic leukemia: a population-based report from the Swedish Adult Acute Leukemia Registry. Leukemia..

[23] Jillella AP, Arellano ML, Gaddh M, Langston AA, Heffner LT, Winton EF (2021). Comanagement strategy between academic institutions and community practices to reduce induction mortality in acute promyelocytic leukemia. JCO Oncol Pract.

[24] Tallman MS, Andersen JW, Schiffer CA, Appelbaum FR, Feusner JH, Ogden A (1997). All-trans-retinoic acid in acute promyelocytic leukemia. N Engl J Med.

[25] Fenaux P, Chastang C, Chevret S, Sanz M, Dombret H, Archimbaud E (1999). A randomized comparison of all transretinoic acid (ATRA) followed by chemotherapy and ATRA plus chemotherapy and the role of maintenance therapy in newly diagnosed acute promyelocytic leukemia. The European APL Group. Blood..

[26] Sanz MA, Martín G, González M, León A, Rayón C, Rivas C (2004). Risk-adapted treatment of acute promyelocytic leukemia with all-trans-retinoic acid and anthracycline monochemotherapy: a multicenter study by the PETHEMA group. Blood.

[27] Sanz MA, Montesinos P, Vellenga E, Rayón C, de la Serna J, Parody R (2008). Risk-adapted treatment of acute promyelocytic leukemia with all-trans retinoic acid and anthracycline monochemotherapy: long-term outcome of the LPA 99 multicenter study by the PETHEMA Group. Blood.

[28] Adès L, Chevret S, Raffoux E, de Botton S, Guerci A, Pigneux A (2006). Is cytarabine useful in the treatment of acute promyelocytic leukemia? Results of a randomized trial from the European Acute Promyelocytic Leukemia Group. J Clin Oncol.

[29] Burnett AK, Kell WJ, Goldstone AH, Milligan D, Hunter A, Prentice AG (2006). The addition of gemtuzumab ozogamicin to induction chemotherapy for AML improves disease free survival without extra toxicity: preliminary analysis of 1115 patients in the MRC AML15 trial. Blood.

[30] Soignet SL, Frankel SR, Douer D, Tallman MS, Kantarjian H, Calleja E (2001). United States multicenter study of arsenic trioxide in relapsed acute promyelocytic leukemia. J Clin Oncol.

[31] Zhao WL, Chen SJ, Shen Y, Xu L, Cai X, Chen GQ (2001). Treatment of acute promyelocytic leukemia with arsenic trioxide: clinical and basic studies. Leuk Lymphoma..

[32] Ghavamzadeh A, Alimoghaddam K, Ghaffari SH, Rostami S, Jahani M, Hosseini R (2006). Treatment of acute promyelocytic leukemia with arsenic trioxide without ATRA and/or chemotherapy. Ann Oncol..

[33] Mathews V, George B, Lakshmi KM, Viswabandya A, Bajel A, Balasubramanian P (2006). Single-agent arsenic trioxide in the treatment of newly diagnosed acute promyelocytic leukemia: durable remissions with minimal toxicity. Blood.

[34] George B, Mathews V, Poonkuzhali B, Shaji RV, Srivastava A, Chandy M (2004). Treatment of children with newly diagnosed acute promyelocytic leukemia with arsenic trioxide: a single center experience. Leukemia.

[35] Ghavamzadeh A, Alimoghaddam K, Rostami S, Ghaffari SH, Jahani M, Iravani M (2011). Phase II study of single-agent arsenic trioxide for the front-line therapy of acute promyelocytic leukemia. J Clin Oncol..

[36] Giannì M, Koken MH, Chelbi-Alix MK, Benoit G, Lanotte M, Chen Z (1998). Combined arsenic and retinoic acid treatment enhances differentiation and apoptosis in arsenic-resistant NB4 cells. Blood.

[37] Zheng PZ, Wang KK, Zhang QY, Huang QH, Du YZ, Zhang QH (2005). Systems analysis of transcriptome and proteome in retinoic acid/arsenic trioxide-induced cell differentiation/apoptosis of promyelocytic leukemia. Proc Natl Acad Sci USA.

[38] Wang G, Li W, Cui J, Gao S, Yao C, Jiang Z (2004). An efficient therapeutic approach to patients with acute promyelocytic leukemia using a combination of arsenic trioxide with low-dose all-trans retinoic acid. Hematol Oncol.

[39] Estey E, Garcia-Manero G, Ferrajoli A, Faderl S, Verstovsek S, Jones D (2006). Use of all-trans retinoic acid plus arsenic trioxide as an alternative to chemotherapy in untreated acute promyelocytic leukemia. Blood.

[40] Ravandi F, Estey E, Jones D, Faderl S, O'Brien S, Fiorentino J (2009). Effective treatment of acute promyelocytic leukemia with all-trans-retinoic acid, arsenic trioxide, and gemtuzumab ozogamicin. J Clin Oncol..

[41] Lo-Coco F, Avvisati G, Vignetti M, Thiede C, Orlando SM, Iacobelli S (2013). Retinoic acid and arsenic trioxide for acute promyelocytic leukemia. N Engl J Med.

[42] Platzbecker U, Avvisati G, Cicconi L, Thiede C, Paoloni F, Vignetti M (2017). Improved outcomes with retinoic acid and arsenic trioxide compared with retinoic acid and chemotherapy in non-high-risk acute promyelocytic leukemia: final results of the randomized Italian-German APL0406 trial. J Clin Oncol.

[43] Cicconi L, Platzbecker U, Avvisati G, Paoloni F, Thiede C, Vignetti M (2020). Long-term results of all-trans retinoic acid and arsenic trioxide in non-high-risk acute promyelocytic leukemia: update of the APL0406 Italian-German randomized trial. Leukemia.

[44] Burnett AK, Russell NH, Hills RK, Bowen D, Kell J, Knapper S (2015). Arsenic trioxide and all-trans retinoic acid treatment for acute promyelocytic leukaemia in all risk groups (AML17): results of a randomised, controlled, phase 3 trial. Lancet Oncol.

[45] Iland HJ, Collins M, Bradstock K, Supple SG, Catalano A, Hertzberg M (2015). Use of arsenic trioxide in remission induction and consolidation therapy for acute promyelocytic leukaemia in the Australasian Leukaemia and Lymphoma Group (ALLG) APML4 study: a non-randomised phase 2 trial. Lancet Haematol.

[46] Abaza Y, Kantarjian H, Garcia-Manero G, Estey E, Borthakur G, Jabbour E (2017). Long-term outcome of acute promyelocytic leukemia treated with all-trans-retinoic acid, arsenic trioxide, and gemtuzumab. Blood..

[47] Lancet JE, Moseley A, Komrokji RS, Coutre SE, DeAngelo DJ, Tallman MS (2016). ATRA, arsenic trioxide (ATO), and gemtuzumab ozogamicin (GO) is safe and highly effective in patients with previously untreated high-risk acute promyelocytic leukemia (APL): final results of the SWOG/Alliance/ECOG S0535 trial. Blood.

[48] Coutre SE, Othus M, Powell B, Willman CL, Stock W, Paietta E (2014). Arsenic trioxide during consolidation for patients with previously untreated low/intermediate risk acute promyelocytic leukaemia may eliminate the need for maintenance therapy. Br J Haematol.

[49] Avvisati G, Lo-Coco F, Paoloni FP, Petti MC, Diverio D, Vignetti M (2011). AIDA 0493 protocol for newly diagnosed acute promyelocytic leukemia: very long-term results and role of maintenance. Blood..

[50] Au WY, Tam S, Fong BM, Kwong YL (2008). Determinants of cerebrospinal fluid arsenic concentration in patients with acute promyelocytic leukemia on oral arsenic trioxide therapy. Blood.

[51] Au WY, Tam S, Kwong YL (2008). Entry of elemental arsenic into the central nervous system in patients with acute promyelocytic leukemia during arsenic trioxide treatment. Leuk Res..

[52] Yilmaz M, Naqvi K, Ravandi F (2019). Current and emerging treatments for acute promyelocytic leukemia. Expert Opin Orphan Drugs.

[53] Sanz MA, Montesinos P (2014). How we prevent and treat differentiation syndrome in patients with acute promyelocytic leukemia. Blood..

[54] Sanz MA, Fenaux P, Tallman MS, Estey EH, Löwenberg B, Naoe T (2019). Management of acute promyelocytic leukemia: updated recommendations from an expert panel of the European LeukemiaNet. Blood..

[55] Montesinos P, Bergua JM, Vellenga E, Rayón C, Parody R, de la Serna J (2009). Differentiation syndrome in patients with acute promyelocytic leukemia treated with all-trans retinoic acid and anthracycline chemotherapy: characteristics, outcome, and prognostic factors. Blood..

[56] Chamoun K, Kantarjian HM, Wang X, Naqvi K, Aung F, Garcia-Manero G (2019). Unrecognized fluid overload during induction therapy increases morbidity in patients with acute promyelocytic leukemia. Cancer..

[57] Daver N, Kantarjian H, Marcucci G, Pierce S, Brandt M, Dinardo C (2015). Clinical characteristics and outcomes in patients with acute promyelocytic leukaemia and hyperleucocytosis. Br J Haematol.

[58] Douer D, Zickl L, Schiffer CA, Appelbaum FR, Feusner JH, Shepherd LE (2011). Late relapses following all-trans retinoic acid for acute promyelocytic leukemia are uncommon, respond well to salvage therapy and occur independently of prognostic factors at diagnosis: long-term follow-up of North American Intergroup Study I0129. Blood.

[59] Shen ZX, Chen GQ, Ni JH, Li XS, Xiong SM, Qiu QY (1997). Use of arsenic trioxide (As2O3) in the treatment of acute promyelocytic leukemia (APL): II. Clinical efficacy and pharmacokinetics in relapsed patients. Blood.

[60] Kwong YL, Au WY, Chim CS, Pang A, Suen C, Liang R (2001). Arsenic trioxide- and idarubicin-induced remissions in relapsed acute promyelocytic leukaemia: clinicopathological and molecular features of a pilot study. Am J Hematol.

[61] Raffoux E, Rousselot P, Poupon J, Daniel MT, Cassinat B, Delarue R (2003). Combined treatment with arsenic trioxide and all-trans-retinoic acid in patients with relapsed acute promyelocytic leukemia. J Clin Oncol..

[62] Aribi A, Kantarjian HM, Estey EH, Koller CA, Thomas DA, Kornblau SM (2007). Combination therapy with arsenic trioxide, all-trans retinoic acid, and gemtuzumab ozogamicin in recurrent acute promyelocytic leukemia. Cancer..

[63] Sanford D, Lo-Coco F, Sanz MA, Di Bona E, Coutre S, Altman JK (2015). Tamibarotene in patients with acute promyelocytic leukaemia relapsing after treatment with all-trans retinoic acid and arsenic trioxide. Br J Haematol..

[64] Beaumont M, Sanz M, Carli PM, Maloisel F, Thomas X, Detourmignies L (2003). Therapy-related acute promyelocytic leukemia. J Clin Oncol..

[65] Pulsoni A, Pagano L, Lo Coco F, Avvisati G, Mele L, Di Bona E (2002). Clinicobiological features and outcome of acute promyelocytic leukemia occurring as a second tumor: the GIMEMA experience. Blood..

[66] Dayyani F, Kantarjian H, O'Brien S, Pierce S, Jones D, Faderl S (2011). Outcome of therapy-related acute promyelocytic leukemia with or without arsenic trioxide as a component of frontline therapy. Cancer..

[67] Braun T, Cereja S, Chevret S, Raffoux E, Beaumont M, Detourmignies L (2015). Evolving characteristics and outcome of secondary acute promyelocytic leukemia (APL): a prospective analysis by the French-Belgian-Swiss APL group. Cancer.

[68] Kumana CR, Au WY, Lee NS, Kou M, Mak RW, Lam CW (2002). Systemic availability of arsenic from oral arsenic-trioxide used to treat patients with hematological malignancies. Eur J Clin Pharmacol.

[69] Au WY, Kumana CR, Kou M, Mak R, Chan GC, Lam CW (2003). Oral arsenic trioxide in the treatment of relapsed acute promyelocytic leukemia. Blood.

[70] Gill H, Kumana CR, Yim R, Hwang YY, Chan T, Yip SF (2019). Oral arsenic trioxide incorporation into frontline treatment with all-trans retinoic acid and chemotherapy in newly diagnosed acute promyelocytic leukemia: a 5-year prospective study. Cancer.

[71] Ravandi F, Koumenis I, Johri A, Tallman M, Roboz GJ, Strickland S (2020). Oral arsenic trioxide ORH-2014 pharmacokinetic and safety profile in patients with advanced hematologic disorders. Haematologica..

[72] Iland JR, J Reynolds, A Boddy, L Khoo, S Yuen, C Bryant, et al. A comparative bioavailability study of encapsulated oral arsenic trioxide and intravenous arsenic trioxide in patients with acute promyelocytic leukemia undergoing consolidation therapy. EHA Abstract PS1028. 2019.

[73] Zhu HH, Wu DP, Jin J, Li JY, Ma J, Wang JX (2013). Oral tetra-arsenic tetra-sulfide formula versus intravenous arsenic trioxide as first-line treatment of acute promyelocytic leukemia: a multicenter randomized controlled trial. J Clin Oncol.

[74] Zhu HH, Huang XJ (2014). Oral arsenic and retinoic acid for non-high-risk acute promyelocytic leukemia. N Engl J Med.

[75] Zhu HH, Liu YR, Jia JS, Qin YZ, Zhao XS, Lai YY (2018). Oral arsenic and all-trans retinoic acid for high-risk acute promyelocytic leukemia. Blood.

[76] Zhu HH, Wu DP, Du X, Zhang X, Liu L, Ma J (2018). Oral arsenic plus retinoic acid versus intravenous arsenic plus retinoic acid for non-high-risk acute promyelocytic leukaemia: a non-inferiority, randomised phase 3 trial. Lancet Oncol.

[77] Yang MH, Wan WQ, Luo JS, Zheng MC, Huang K, Yang LH (2018). Multicenter randomized trial of arsenic trioxide and Realgar-Indigo naturalis formula in pediatric patients with acute promyelocytic leukemia: Interim results of the SCCLG-APL clinical study. Am J Hematol.

